# Case Study on the Maintenance of a Construction Monitoring Using USN-Based Data Acquisition

**DOI:** 10.1155/2014/879308

**Published:** 2014-07-06

**Authors:** Sangyong Kim, Yoonseok Shin, Gwang-Hee Kim

**Affiliations:** ^1^School of Construction Management and Engineering, University of Reading, Whiteknights, P.O. Box 219, Berkshire, Reading RG6 6AW, UK; ^2^Department of Plant/Architectural Engineering, Kyonggi University, 152-42 Gwanggyosan-ro, Yeongtong-gu, Suwon-si, Gyeonggi-do, 443-760, Republic of Korea

## Abstract

In recent years, there has been an increasing interest in the adoption of emerging ubiquitous sensor network (USN) technologies for instrumentation within a variety of sustainability systems. USN is emerging as a sensing paradigm that is being newly considered by the sustainability management field as an alternative to traditional tethered monitoring systems. Researchers have been discovering that USN is an exciting technology that should not be viewed simply as a substitute for traditional tethered monitoring systems. In this study, we investigate how a movement monitoring measurement system of a complex building is developed as a research environment for USN and related decision-supportive technologies. To address the apparent danger of building movement, agent-mediated communication concepts have been designed to autonomously manage large volumes of exchanged information. In this study, we additionally detail the design of the proposed system, including its principles, data processing algorithms, system architecture, and user interface specifics. Results of the test and case study demonstrate the effectiveness of the USN-based data acquisition system for real-time monitoring of movement operations.

## 1. Introduction

With the increasing demands of skyscraper, airport hub, and rail network development, the scale of construction projects has significantly grown. These large-scale construction projects involve complex interfaces for which the development of construction safety monitoring has become considerably important [1]. After completion of a construction project, the sustainability of the construction site continues to play a crucial role in safety assessment through structural health monitoring (SHM). In addition, damage to humans is a more serious situation that can occur from unpredictable factors such as natural disasters and safety ignorance. The fact of human damage and its economic costs underscores the importance of continual monitoring of construction activities. The introduction of SHM can enable the rational coping with primary damage through objective SHM data [2]. This important SHM trend in construction requires rational methods to ensure health and safety (HS) and effective maintenance control in the face of uncertainties and associated risks. Therefore, installation of automated monitoring systems in current projects is required for identifying the state of building change. This is because it is impossible to implement timely checks and repairs of facilities using traditional methods of passive monitoring systems. For example, monitoring results of valuable data collected from construction sites are not applied in a timely manner if the project administrator does not have sufficient data processing speed [3].

To overcome current limitations, construction monitoring technologies have been rapidly developing with the advancement of information technology (IT) and telecommunications [4]. Automatic monitoring systems can obtain in real time accurate information of ground movement analyses and impact assessments of nearby building conditions [5]. These procedures of gathering, processing, and analyzing of recorded experimental data accumulated from the field are expected to improve the precision and reliability of results while enabling rapid coping with cracks and vibrational problems. The advent of the ubiquitous sensor network (USN) approach was prompted by the absence of suitable communication for the timely acquisition of construction resources in large-scale projects [6–8]. The USN system improves HS monitoring of a suspension building. Therefore, the use of new technologies, such as tablets and smart phones, has been adopted to expedite the monitoring process. These technologies contain long-term evolution (LTE) technology to connect in real time the main servers with multiple devices. Accordingly, LTE technology can enable effective data transfer from a remote location to the main office. The related software applications, specifically collection static data (CSD) and the integration measuring system (IMS), can maximize the advantages of wireless sensor technology. These applications for collecting data are web-based; therefore, monitoring is available from any location with a wireless Internet connection [9]. Through the various calibration tests mentioned above, monitoring instruments can enhance monitoring reliability and durability. In addition, the universalization of monitoring instruments can automatically reduce monitoring system installation costs.

The objective of the present research is to report the necessity of automated monitoring systems for enhancing sustainable construction maintenance and to ensure HS. The following objectives must be achieved to meet the stated aim. (1) Obtain more accurate and reliable monitoring data, which would enable easier and more effective analysis. To this end, the implementation of automated monitoring systems should be considered for practical construction sites. (2) Enable monitoring for the smooth implementation of the automatic monitoring system and improve economic feasibility assessments to accelerate the implementation.

## 2. Literature Review

Many studies have been carried out to reduce the gap between predictions and real situations. Various IT application approaches have been proposed for construction monitoring through a diversity of projects. These include equipment monitoring with a global positioning system (GPS), material tracking with radio frequency identification (RFID) devices, and wireless networking technologies [2, 10]. However, previous studies have identified several limitations of GPS and RFID techniques. For one, GPS technology can be affected by lack of signals due to geographic factors and weather. Other GPS limitations include poor GPS signals, loss of GPS signal integrity, and limited positioning accuracy [11]. Additionally, RFID has reliability problems because the RFID sensors cannot automatically distinguish between a failed sensor and the nonoccurrence of an expected event [2, 10]. For the above reasons, development of a wireless network system would address the need for fully automatic monitoring in construction sites. As a potential solution, mobile technology has nevertheless presented major challenges in the IT domain of construction projects [12]. The diversity of many possible implementation scenarios involves HS applications, asset tracking logistics, building monitoring, and provisioning of equipment maintenance information [13]. However, the current deployment of wireless network systems focuses on static information delivery without considering user satisfaction. The consideration of user satisfaction, such as user profiles, role preferences, and construction tasks, should be addressed to ensure the efficacy and accuracy of information delivery during the construction process, thereby saving valuable time. Potential benefits can then be expected, such as improvement in efficiency and productivity [14, 15].

The initiative of primary construction maintenance monitoring began with the Committee of Sponsoring Organization of the Treadway Commission (COSO). According to COSO, continuous monitoring enables management to continually automate the review of business processes to ensure effective performance. Automated business processes can involve assessing the effectiveness of controls and detecting associated risk issues. Additionally, improved performance can encourage adherence to compliance standards and increase the cost-effectiveness of controls and monitoring through IT solutions. Woo [3] proposed the estimating of circumstances across all construction activities through general construction monitoring and maintenance monitoring. General construction monitoring compensates for the uncertainty of the design and provides safety and economic feasibility by validating the design. Maintenance monitoring serves to consistently check HS for existing building issues and to maintain optimum conditions. Maintenance monitoring can contribute to valuable economic maintenance of construction facilities through objective and effective data. These benefits can improve management of the entire process of increasingly complex construction projects currently constrained under the inadequacy of traditional monitoring systems. Therefore, continuous maintenance monitoring becomes increasingly more important for improving the whole construction performance in terms of safety elements such as checking structural differential movement and rotation, cracking, and building vibration. However, existing monitoring systems, which manually aggregate productivity reports from sites, are unable to disseminate information in real time from both sites and office management with sufficiently detailed information.

## 3. Methodology

In this paper, we explain the application of automatic monitoring system methods that employ portable data loggers and wired and wireless systems. In addition, we outline the structure of these connection methods. The application of these methods considers the distance between the office and construction sites and conditions such as underground work, weather, and the number of workers. Additionally, appropriate measuring sensors, such as the EL-beam, cracking test machines, and vibrational measuring devices, are installed in construction sites according to characteristics of the given construction project. We herein use a case study to explain the operation of CSD and IMS programs. The case study involves the practical application of an automatic monitoring system for a complex building in South Korea. The case study is a qualitative method supported by literature review to substantiate the necessity of construction monitoring and case study content analysis.

## 4. Comparison of Existing Monitoring Systems

Existing monitoring methods at construction sites involve either passive or automated monitoring depending on the construction condition. The selection of the method is directly related to economic aspects. The operation of existing automated monitoring systems is marked by inadequate technical expertise with lack of issue recognition [4]. The primary use of the automated monitoring system requires significant investment to implement it. However, in the long term, it is much more economically feasible than existing monitoring systems for projects and complex construction environments. The automated system can notify administrators with an alarm feature about changeable situations to enable immediate analysis. These advantages, which are readily reflected in the ongoing construction process, make automated monitoring systems far superior to existing monitoring systems.

In Table 1, the comparison of existing and automated monitoring systems is summarized. Although existing monitoring systems continue to be used in construction sites because of their time-efficient installation and easy maintenance benefits, they are nevertheless time consuming in terms of analyzing accumulated data with high human resource constraints. Furthermore, existing monitoring is marked by low precision and the difficulty of monitoring that is dependent on construction conditions. These limitations, however, can be reduced through automated monitoring systems because the latter systems enable control in varying the frequency of monitoring and generating its data while providing precise data, time savings, and more efficient use of human resources.

## 5. Integration of Information Services

### 5.1. System Processes

**Figure 1:**
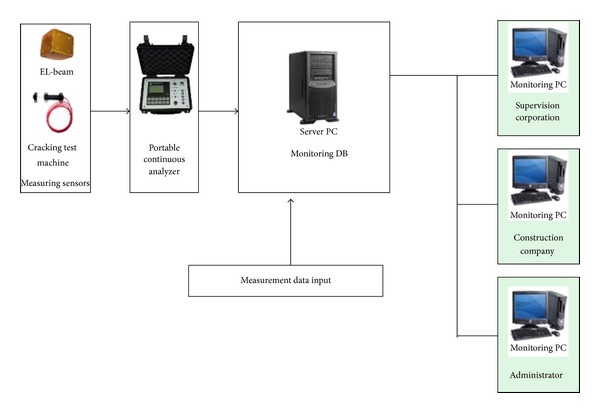
Flowchart of portable data logger method monitoring.

Project managers require a robust monitoring system that can ensure that the most current information is delivered and represented in a timely and comprehensive manner, thereby enabling control decisions to be made as quickly and easily as possible.  Figure 1 depicts the flow of a monitoring system as a portable, continuous analyzer through a data logging method. The automated system consists of a sensor, data logger, multiplexer, control cable, and computer. In addition, it includes a power supply for the data logger and multiplexer. The integrated system handles general processing for measurements. The computer-stored data is sent to a central database or a web server at specific time intervals based on a previous setting. The network system is configured to support remote control and data access from any remote area over the Internet. The information is made available to recipients in real time on websites as raw data and graphs. The data logger saves the measurement data in memory and sends a warning or wireless call when the data exceeds the limit set in the integrated system. After measurements are performed with the portable data logging equipment, the measurement data is entered in a measurement system in the main computer to provide access to it.

In the case of a wired connection, signal cables are used to immediately save data into an automated measurement program on an office computer for real-time observation. If a field management limit is specified in the program, the program activates a notification sound and warning screen if any entered data exceeds the set limit. This provides users with easy notifications without monitoring. The monitoring system sends safety measurement data at specific intervals to the computer for access. The data logger wirelessly communicates in real time with the remote control monitoring data to enable identification of the problem and the appropriate action.

The continuous analyzer device primarily saves the collected data. The analyzer and LTE are connected by the RS232 communication method. The onsite continuous analyzer device and remotely installed wireless communication device can exchange measurement data through LTE communication. The measurement data collecting server can analyze the data gathered by the continuous analyzer device and process and save it for administrators to view. Lastly, the collecting server is comprised of a database server and web server. The database server backs up the saved data, while the web server ensures online data access from any location.  Table 2 summarizes the comparison of CSD and IMS programs. Based on this information, which includes a comparison of their respective strengths and weaknesses, the CSD program is appropriate in a measurement system during construction, whereas IMS is suitable for the maintenance monitoring system.

### 5.2. Monitoring System Diagram

**Figure 2:**
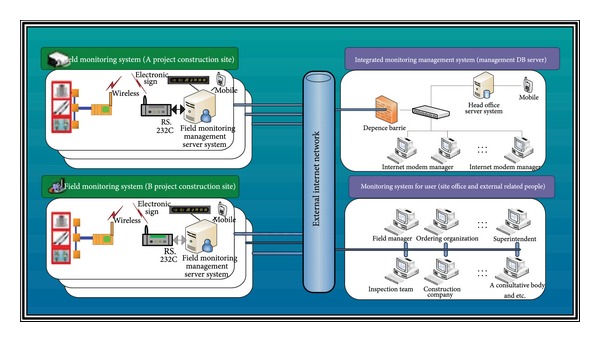
The overall diagram of monitoring system.

In Figure 2, the overall structure of the automated monitoring system is presented. For Project A and Project B of the different construction sites, field measuring sensors are installed in the construction structure. In addition, a field measuring system is used to wirelessly communicate the measured data from the measuring sensors to the field office. Therefore, these project sites can send organized data from construction sites to the head office and external related organizations such as inspection teams. Moreover, superintendents and consultants can obtain the organized data over an external network. Security measures are in place to protect the data from viruses.

**Figure 3:**
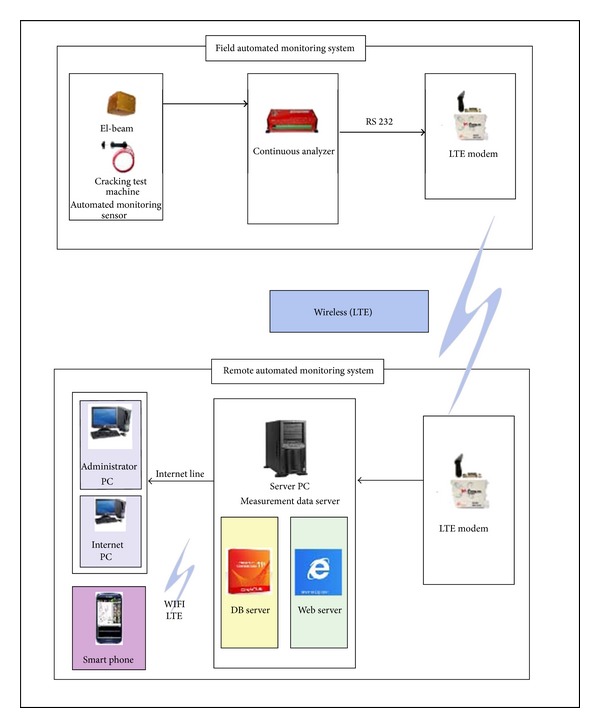
The diagram of applied monitoring system (LTE).

Figure 3 outlines the process by which the onsite monitoring system is wirelessly connected to the remote automated monitoring system. Automated monitoring sensors are installed in the construction site to provide continuous monitoring; the data logger connects to the monitoring sensors by signal cable. This data logger, a continuous analyzer, is an electronic device that records data over time or in relation to locations either with an integrated instrument sensor or through external instrument sensors. The continuous analyzer can operate on any computer platform by simple instructions. In addition, it can collect in real time the measured data from construction sites over the Internet. Various sensors measure voltage, resistance, frequency, and input/output of digital signals. The measured data, which can transfer engineering numerical values, is comprised of linearization results from the general temperature sensor. The measured data analysis process is simple and software, such as Lotus and Excel, can be used. The collected measured data is immediately presented on the screen of the continuous monitoring analyzer and administrator computer for saving. The continuous analyzer can provide reduced maintenance costs due to less power consumption. Moreover, it can be powered by a small portable battery for long time periods in construction sites, where it can perform network, alarm, and indicating graph functions.

### 5.3. Program Components

**Figure 4:**
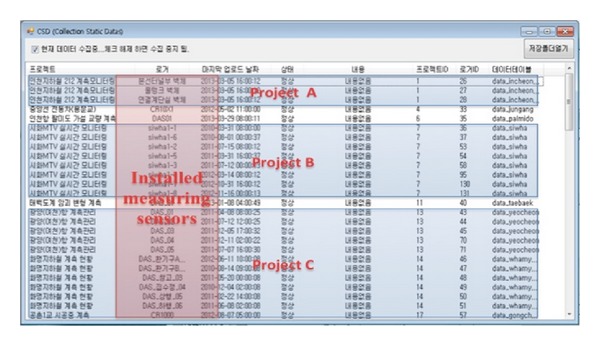
Collection static data program.

The CSD program is composed of the CSD function and general CSD without the function.  Figure 4 depicts the CSD program used for different construction sites. The red color box in the program denotes sites where sensors are installed.

**Figure 5:**
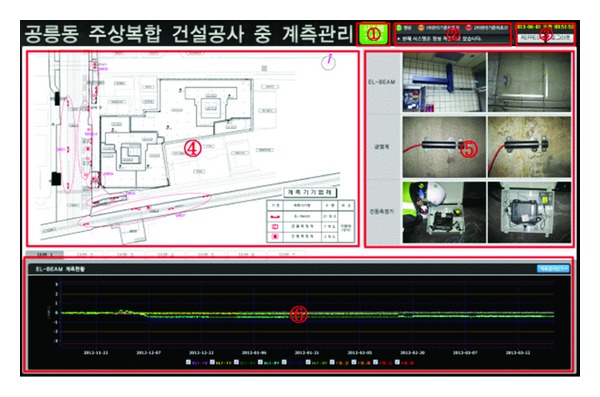
The example of CSD.

If one of the lists is selected, the screen appears, as shown in Figure 5. The CSD program screen is comprised of six components.

1. System diagram → ①
2. Current condition → ②
3. Login/logout → ③
4. Location of field measuring equipment → ④
5. Installation pictures → ⑤
6. Graphical visualization of real-time data monitoring → ⑥

**Figure 6:**
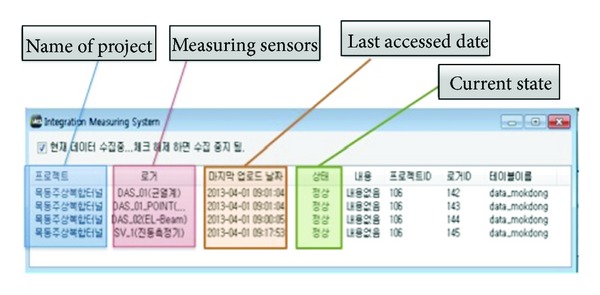
Integration measuring system (IMS) program.

Figure 6 illustrates the program implemented for IMS. It is comprised of the project name, measuring sensors, the last accessed date, and the current state. If the project is selected, the program will launch.

## 6. Case Study

We now consider a case study and demonstrate how the USN environment can help personnel from different functional groups conduct collaborations. For the case study, the Gongneung-dong complex building project from the apartment industry sector of Hyundai Amco Construction Ltd. was selected. The project was a complex building located at 670-20 Gongeung-dong, Nowon-gu, Seoul, South Korea. It was composed of two buildings with 36 stories and exclusive use of the apartments. The total lot area was 84 m^2^ out of 6,026.50 m^2^, as shown in Table 3.

### 6.1. Experimental Field Setup

The field measuring instruments used in the case study included the EL-beam, cracking test machine, and vibration measurement apparatus, as shown in the number ⑤ of Figure 5.

The EL-beam sensors monitored differential movement and rotation in the structures. The horizontal beam sensors monitored settlement and heave. The vertical beam sensors monitored lateral displacement and deformation. These two methods of installation enabled monitoring of structural behavior under loads as well as the stability of the structures. Stabilization measures were then provided. The EL-beam operates by means of a beam sensor consisting of an electrolytic tilt sensor—a precision bubble level electrically sensitized as a resistance bridge—that is attached to a rigid metal beam. The beam, typically one to two meters long, was mounted on anchor bolts set into the structure. Structural movement changes the tilt of the beam and the sensor output. The cracking test machine then evaluates progressive cracking in the walls. The results of the cracking test can help identify the appropriate time to perform repairs based on changes of crack width. It can be affected by temperature changes, crack shapes, and various construction loads, such as superimposed loads and movement of construction equipment. Therefore, continuous vibration monitoring can provide information by which building health conditions can be assessed. Lastly, vibration can be simply defined as the cyclic or oscillating motion of a machine or machine components from a position of rest. Construction vibration can be generated from various forces, such as the movement of large-scale equipment and progressive work packages. With the results of direction change over time, the analysis enables breakdown maintenance and scheduled or preventative maintenance. Furthermore, the trending and analysis of vibration performance helps identify developing problems before failure and extensive damage occur, thereby providing predictive maintenance.

### 6.2. Monitoring System Application

**Figure 7:**
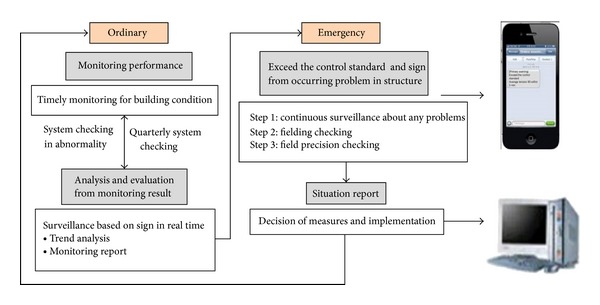
Warming system (SMS service).

The current state is indicated on the computer screen through the main monitoring sensors. The information can be obtained from saved data in the computer or by directly accessing the field measuring machine. The data can be analyzed with various methods such as plain text, Excel, and graphs. The current monitoring state is automatically updated every ten minutes. The time interval enables the setting according to the user and administrator. The function of the main monitoring sensor is to issue a notification on the computer screen and mobile phone with the integral short message service (SMS) when the standard is exceeded (Figure 7). Because this monitoring system is web-based, it can be used in any location with a computer and Internet access. If the monitoring program is checked on the web browser, the CSD program is first initiated and the web browser accesses the web-based system. From that point, it can verify the monitoring program after entering the ID and password on the login page.

**Figure 8:**
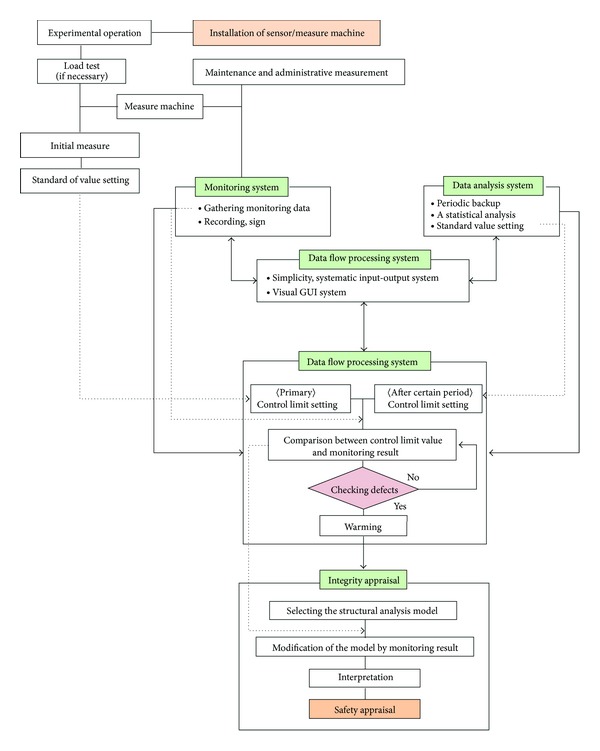
The diagram of monitoring system.

The key flow of the measurement monitoring system is summarized in Figure 8. The collected information from field monitoring sensors and machines are sent to the monitoring system in a construction site. This information can be shared between the construction site and remote locations. The monitoring system can regularly record collected data through a backup process. In addition, it can perform statistical analysis through the information analysis system in the data flow processing system. This system can then issue notifications to the administrator, depending on the standard of value and the set limitation. Using this systematic procedure, the project is safety evaluated based on the result.

### 6.3. CDM Application Program

In the CDM application, the following three elements can be presented by selecting the project system structure: current monitoring condition, administrator login, and installed measuring sensors located in the construction site.

#### 6.3.1. Project System Structure

**Figure 9:**
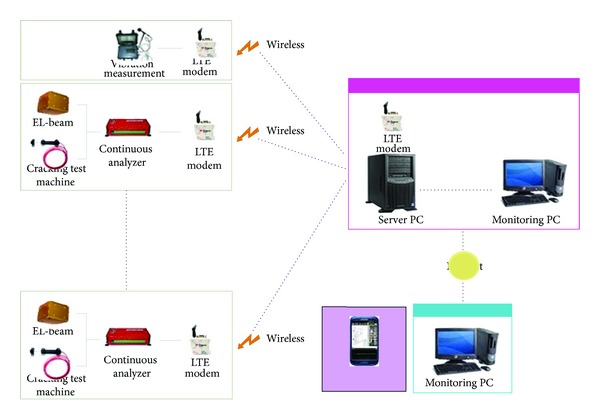
The system structure.

The system structure diagram is comprised of field measurement equipment that includes installed EL-beams, cracking test machines, and vibration measuring devices in a construction site along with a wireless LTE modem. Information gathered from all field measurement equipment is sent by the LTE modems. These modems then connect to the main server in a remote location. Therefore, administrators or users can identify the measurement information gathered from a construction site by connecting to the Internet. At that point, they log into the CDM monitoring program (Figure 9).

#### 6.3.2. Location Map of Installed Measuring Sensors in the Construction Site

The number ④ of Figure 5 depicts in a cross-sectional view (of every floor) the locations where the EL-beam, cracking test machine, and vibrational measuring device are installed in the building. The table located at the bottom-right side outlines the number of sensors with three different kinds of logistic in the construction site.

#### 6.3.3. Graphical Visualization of Real-Time Data Monitoring

The graphical visualization of real-time data monitoring, as shown in the number ⑥ of Figure 5, is located at the bottom of the program. It depicts the data in real time with the maintenance standard. The top of the image is divided into cross-sectional views of every floor in the building so that it can communicate the data in a timely manner when monitoring is needed in a specific place in the building.

**Figure 10:**
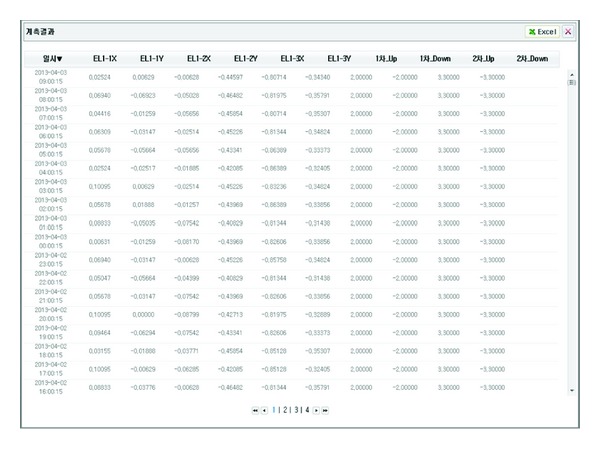
Saving function and saved result lists through MS Excel.

In addition, the project status can be viewed using a simple web browser, as shown in Figure 10. The status view presents the data log extracted from monitoring in which the read time at ten-minute intervals is displayed. Moreover, it is possible to save the measurement data and open files of previously saved results with Excel (Figure 10).

## 7. Result and Discussion

Timely field monitoring can resolve the gap between predictions and real situations by enabling the analyzing of construction validity. Monitoring by USN-based data acquisition can promote construction safety through the analyzing of collected data. Use of this data supports effective decisions and suggestions for effective methods in terms of safety maintenance and design changes. The automated monitoring system can provide an improvement of HS based on its timely acquisition of data. In addition, it enables the establishment of countermeasures for the HS and ensures integration with building maintenance in contribution to construction management. Maintenance monitoring contributes effective and economic maintenance for construction facilities by generating objective and effective data. These benefits can improve management of the entire process of increasingly complex construction projects currently constrained under the inadequacy of traditional monitoring systems. Therefore, continuous maintenance monitoring becomes ever more important for improving the whole construction performance in terms of safety elements, such as checking structural differential movement and rotation, cracking, and building vibration.

However, the above applications consider economic aspects in utilizing portable devices and analysis software. This is because the initial cost of installation, such as for the server construction and field experimental setting, would be expensive. In addition, the automated monitoring system is not only installed with a high investment, but basic limitations remain in terms of monitoring instruments and malfunctions caused by external factors, such as temperature and weather, during the construction and maintenance period.

## 8. Conclusions and Recommendations

With the advance of IT applications, all project participants, such as clients, construction companies, and inspection teams, expect smooth communication because the standardized measurement data can be rapidly accessed using the Internet and portable devices. This enables the transformation of the work environment, which was previously a vertical business relationship in which it was difficult to share information.

In this paper, we introduced monitoring equipment, such as a continuous analyzer, and measuring instruments, such as the EL-beam, cracking test machines, and vibration measuring devices for operating automated monitoring systems. Furthermore, we explained the methods of data connection and the technology of information processing. The advantages of construction industry efficiencies reduced construction time and a cost-effective integrated monitoring program using the Internet yield potential for the growth of construction technology. After completion of the network, the use of tablets and other personal devices enable the easy and timely performance of processes by administrators and managers in multiple construction sites.

An integrated operation program that facilitates the integration of construction databases and measured data processed in real time requires further development to achieve the advancement of an automated monitoring system. Such a program could enhance operational reliability through appropriate use of analysis systems in construction sites and consideration of building state assessments. Higher quality data reliability enables easy construction monitoring for every participant in the project using an installed network. Therefore, the installation can reduce system costs through the development of an integrated program.

## Figures and Tables

**Table 1:** Comparison between existing monitoring and automated monitoring system.

	Automated monitoring	Existing monitoring
Advantages	(i) High precision (ii) Enable to control various frequencies of monitoring (iii) Economy of time for monitoring analysis to immediately response (iv) Many field experimental machines work at the same time (v) Enable to output the data with monitoring (vi) Saving the manpower	(i) Economy of time for installation (ii) Enable to analyze monitoring in the construction site (iii) Easy maintenance

Disadvantages	(i) Expensive material cost (ii) Time consuming to install the automatic system (iii) Requiring field observation	(i) High manpower consumption (ii) Low precision (iii) Level of difficulty depends on the field condition (iv) Time consuming for data analyzing (v) Unavailable measurements in terms of bad weather

**Table 2:** The comparison between CSD and IMS.

	CSD	IMS
Strength	(i) Simple system configuration (ii) Possibility to develop an installation program and web program	(i) As operated on the web base, monitoring check is possible anywhere with Internet connection (ii) Possible to process data in diverse formats such as report and graphs (iii) Possible to set user authority (iv) Possible to customize programs according to specific on-site conditions

Weakness	(i) Program use faded down (ii) Slow loading speed when data accumulation is huge	System installation is costly

**Table 3:** The project summary.

Location	670-20 Gongneung-dong, Nowon-gu, Seoul, South Korea
Total lot area	6,026.50 m^2^
Type of building	Complex building
Number of stories	
Basement	5th
Stories	36th
Gross area	53,489.1066 m^2^
Building coverage ratio	59.51%
Floor area ratio	587.72%
Exclusive use of apartment	84 m^2^/2 buildings (A, B)
Total house holds	234 (A-118/B-116)
